# Top-Cited Articles on Dysphagia and Cognitive Impairment: A Scopus-Based Bibliometric Analysis of Publications Retrieved Through October 2025

**DOI:** 10.7759/cureus.110620

**Published:** 2026-06-10

**Authors:** Norman-Vivaldi Montúffar-Otero, Annel Gómez-Coello, Paulina Murphy, Aisha-Alexandra Murillo-Chavez, Ernesto Roldan-Valadez

**Affiliations:** 1 Department of Phoniatrics and Speech-Language Pathology, Instituto Nacional de Rehabilitación Luis Guillermo Ibarra Ibarra, Mexico City, MEX; 2 Division of Research, Instituto Nacional de Rehabilitación Luis Guillermo Ibarra Ibarra, Mexico City, MEX; 3 Department of Radiology, I.M. Sechenov First Moscow State Medical University (Sechenov University), Moscow, RUS

**Keywords:** bibliometric analysis, citation metrics, cognitive impairment and dementia, correlates of cognitive impairment, deglutition disorders, dysphagia, neuro-rehab, parkinson' s disease, scoping review, stroke

## Abstract

Deglutition disorders and cognitive impairment co-occur frequently in older adults and in patients with neurodegenerative or cerebrovascular disease, yet the intellectual structure and collaborative patterns at this clinical interface have not been mapped. We performed a bibliometric analysis, with scoping-review-style charting, of publications indexed in the Scopus database through the search date of 10 October 2025; we retrieved 1,190 records and retained the 100 most-cited articles for in-depth analysis. Because the most recent publications (2023-2025) have not yet accrued sufficient citations, the resulting top-100 corpus spans 2009-2022. Articles were stratified into three citation tiers: Hyperclassics (≥500 citations, n = 11), Top-Class (250-499 citations, n = 20), and Classics (100-249 citations, n = 69).

Performance analysis and science mapping were conducted in R version 4.4.2 (R Foundation for Statistical Computing, Vienna, Austria) using the bibliometrix and biblioshiny packages (open-source; https://www.bibliometrix.org/). Continuous variables were compared using the Kruskal-Wallis test followed by post hoc Mann-Whitney U tests; categorical variables with the chi-squared test and Cramér's V; and correlations among journal indicators with Spearman's rho and 95% confidence intervals derived via Fisher's z-transformation. The corpus accumulated 30,872 total citations (range 112-4,312). Stroke was the leading venue (6 articles; 7,909 citations; 25.6% of the corpus), and the United States contributed 32 articles representing 47% of all citations, followed by the United Kingdom, Italy, Canada, and Germany.

Hyperclassics significantly out-cited Classics in both total citations and citations per year (both p<0.0001) but not in author count or active years. Inter-metric correlations among Journal Impact Factor (JIF), CiteScore, SCImago Journal Rank (SJR), and Source Normalized Impact per Paper (SNIP) were uniformly strong (ρ>+0.84; all p<0.001), whereas correlations between these journal-level indicators and article-level citations were weak (ρ +0.22 to +0.29; all p<0.05). Keyword co-occurrence resolved three thematic clusters-stroke, dementia, and dysphagia (clinical burden and nutrition); Parkinson's disease and movement disorders; and neurological symptoms, cognition, and diagnostic imaging-while a complementary thematic-evolution analysis traced eight finer research streams. The field is dominated by stroke-related research and Anglo-American academic medicine, with a small set of influential guidelines driving disproportionate citation weight. Within this most-cited corpus, articles centred explicitly on the dysphagia-cognition interface were comparatively few (14% of the corpus), whereas disease-context and guideline records predominated; sensitivity analyses excluding peripheral records preserved the thematic and geographic structure. Estimating under-representation relative to clinical burden would require a field-normalised denominator and is proposed as future work. Future work should prioritize mechanistic neuroimaging in cognitively impaired patients, validated screening instruments adapted for dementia and parkinsonism, and comparative-effectiveness trials of dysphagia interventions across diagnostic categories.

## Introduction and background

Dysphagia (also termed deglutition disorders) is highly prevalent among the aging population suffering from cognitive impairment, regardless of the nature of the neurodegenerative or cerebrovascular disorders. These disorders are both overlapping and interrelated because cortical and subcortical dysphagia and cognitive functions of attention, executive functions, and awareness are processed by the same systems. As a result, disturbances to this system, as a rule, will ultimately affect all of the mentioned functions at the same time [1-3]. As a result, patients do not have the ability to signal, and cognitive impairment due to dementia, parkinsonism, and impaired cognitive functioning after a stroke, which cascades public healthcare systems into malnutrition, dehydration, aspiration pneumonia, prolonged hospitalization, and mortality [4-6].

The reported prevalence of oropharyngeal dysphagia is estimated to be greater than 80% in moderate to severe Alzheimer’s disease, around 80% in individuals with Parkinson’s disease, occurs in 30-50% of individuals in the first few weeks after a stroke, and is reported in more than 50% of residents of nursing homes, regardless of a formal dementia diagnosis [5-7]. A 2022 meta-analysis showed that dysphagia is a leading cause of pneumonia, malnutrition, and death in the elderly. This demonstrates the public health importance of the issue [4]. Despite the presence of such a burden, the integration of dysphagia screening among cognitively impaired people at individual health service centers is poor and does not vary significantly [8,9].

Neuroimaging has demonstrated that swallowing is a bilateral, distributed process across the cortex. The complete connection and efficiency of this process depend on the affected areas of the insula, operculum, premotor, and basal ganglia, as seen in cognitive disorders [10-13]. Functional MRI and diffusion-tensor imaging have demonstrated that a deterioration of the cortico-bulbar swallowing network can be a predictor of the severity of dysphagia and compensatory dysphagia [14,15]. The data available has shown that of all the studies cited in the field, the 2019 guidelines from the American Heart Association/American Stroke Association in the management of people with acute ischemic stroke clearly mention the screening of dysphagia and place it as a Level I screening recommendation [16], and that influential reviews on Parkinson's-disease dysphagia and gastrointestinal dysfunction have influenced and directed clinical practice worldwide [17,18].

Cognition is mechanistically and clinically intertwined with swallowing. Safe, efficient deglutition depends on attention and alertness to the act of eating, executive control of feeding behaviour, awareness of aspiration risk, and the capacity to learn and adhere to compensatory strategies, and it can be disrupted by apraxia, hemispatial neglect, and impaired interoception. These cognitive contributions differ across conditions: in dementia, and notably in Alzheimer's disease, progressive impairment of attention, memory, and feeding praxis predominates; after stroke, vascular cognitive impairment compounds sensorimotor deficits in the swallowing network; and in Parkinson's disease, executive and attentional dysfunction interact with bradykinetic control of the oropharyngeal musculature. Distinguishing these phenotypes clarifies why a bibliometric map of the dysphagia-cognition interface is clinically useful.

On the diagnostic side, validated bedside instruments such as the Eating Assessment Tool-10 (EAT-10), the Gugging Swallowing Screen (GUSS), and the Volume-Viscosity Swallow Test, together with instrumental gold standards (Fibre-optic Endoscopic Evaluation of Swallowing (FEES) and Videofluoroscopic Swallow Study (VFSS)), have improved case finding, while consensus statements have begun to standardize the approach in cognitively impaired populations [8,9,19,20]. In the therapeutic domain, the PhEAST randomized clinical trial has made a considerable advance in the field of pharyngeal electrical stimulation for post-stroke dysphagia [21], and network meta-analyses have ranked expiratory muscle strength training, repetitive transcranial magnetic stimulation, and chin-tuck-against-resistance as effective non-pharmacological interventions [22,23]. Despite this momentum, no synthesis, to our knowledge, has mapped the intellectual and collaborative architecture of the field at the intersection of deglutition and cognition.

Beyond the traditional systematic review, bibliometric analysis highlights a different perspective. Instead of effect size consolidation, it defines knowledge production, the producers, and the coverage across journals, institutions, and countries [24-26]. Within neuroscience and pharmacology, scoping reviews combined with bibliometric analyses have been particularly useful for advanced or interdisciplinary domains [27-29]. The Top-100 most-cited construct, popularized by Garfield's notion of Citation Classics [30], isolates the papers that have shaped a field's intellectual trajectory and provides a tractable corpus for performance and science-mapping analyses.

The purpose of this study was to perform a bibliometric analysis of the top 100 most-cited articles on swallowing disorders and cognitive deficits (publications retrieved through 10 October 2025) regarding: (i) analyze the patterns of publication and citation; (ii) determine the most cited authors, articles, and the most important journals and institutions in relation to the discipline; (iii) construct the intellectual and associative domain of the discipline with the help of citation, keyword, and authorship networks; and, (iv) highlight the intellectual trajectory and the unexplored terrain to inform the direction of anticipated studies.

## Review

Materials and methods

Search Methodology and Data Sources

Bibliometric analysis (BA) provides a quantitative, reproducible window into the authorship, scholarly productivity, and journal-quality landscape of a defined research domain [24,25]. By supplementing the narrative perspective with citation-based metrics, BA exposes the intellectual and collaborative architecture of a field in ways that traditional reviews cannot [26,28,29]. All data for the present work were drawn from Scopus (https://www.scopus.com; Elsevier, Amsterdam, The Netherlands), accessed on 10 October 2025. Scopus was chosen for its broad, multidisciplinary indexing of biomedical journals and conference proceedings, along with its citation export utilities, which support reproducible, citation-based analyses of research performance and collaboration [24]. Although multi-database retrieval is preferable for systematic reviews aimed at evidence synthesis, single-database (Scopus-only) retrieval is the established approach for top-cited bibliometric and scoping evaluations [27-29].

Journal Impact Factor (JIF) values and quartile rankings (Q1-Q4) were extracted from Clarivate Analytics' Journal Citation Reports (JCR; https://jcr.clarivate.com) to complement Scopus citation counts with independent journal-quality indicators [26,31]. Because this study used only publicly available bibliographic and citation metadata, Institutional Review Board approval was not required.

Software

Data wrangling and descriptive analyses were performed in Microsoft Excel v.16.67 (Microsoft Corporation, Redmond, WA, USA) and in Python v.3.10 with the pandas, numpy, and SciPy libraries. Performance analysis and science mapping were conducted in R version 4.4.2 (R Foundation for Statistical Computing, Vienna, Austria; https://www.r-project.org/) using the bibliometrix and biblioshiny packages (open source; https://www.bibliometrix.org/) [32]. The co-citation, keyword co-occurrence, and co-authorship network were generated in Python version 3.10.12 (Python Software Foundation; https://www.python.org/) [33] using the NetworkX (https://networkx.org/), python-louvain (https://github.com/taynaud/python-louvain), and Matplotlib (https://matplotlib.org/) libraries. A priori thresholds were set at ≥10 citations per author, ≥2 documents per institution, country, or journal, and ≥5 co-occurrences per keyword. Correlation matrices annotated with significance stars, dual-panel world maps, and Gantt-style thematic timelines were produced in Python with matplotlib, seaborn, and networkx.

Compliance with Reporting Standards

This bibliometric analysis adhered to the Preferred Reporting Items for Systematic Reviews and Meta-Analyses Extension for Scoping Reviews (PRISMA-ScR) [34] and, where applicable, to the PRISMA 2020 statement [35].

Inclusion and Exclusion Criteria

We included original research articles and reviews that addressed any form of deglutition disorder (dysphagia) alongside any form of cognitive impairment or neurocognitive disorder, including but not limited to dementia, Alzheimer's disease, Parkinson's disease, and the cognitive sequelae of stroke. To qualify, articles had to be published in peer-reviewed journals, provide complete bibliographic metadata, address the cognition-dysphagia relationship explicitly, and be retrievable through Scopus.

Studies were excluded if they (i) focused exclusively on cancer, oncology, cardiovascular disease, diabetes, or systemic inflammation; (ii) employed animal models or in-vitro designs; (iii) were secondary or tertiary literature (conference papers, books, notes, editorials, short surveys, letters to the editor, or book chapters); (iv) were duplicates; or (v) were not available in English. The complete Boolean search algorithm, organized into four conceptual domains and reproduced verbatim, is provided in Table 1.

**Table 1:** Complete Scopus search algorithm for the bibliometric analysis on dysphagia and cognitive impairment ( TITLE-ABS-KEY ( ( "Deglutition Disorders" OR "Dysphagia" OR "Swallowing Disorders" ) ) AND TITLE-ABS-KEY ( ( "Cognitive Impairment" OR "Cognitive Impairment" OR "Neurocognitive Disorders" OR "Cognitive Decline" ) OR ( "Dementia" OR "Alzheimer’s Disease" OR "Parkinson’s Disease" OR "Stroke" ) ) AND TITLE-ABS-KEY ( ( "Assessment Tools" OR "Diagnostic Criteria" OR "Management Strategies" OR "Treatment" OR "Rehabilitation" ) ) AND NOT TITLE-ABS-KEY ( ( "Animal Models" OR "In Vitro" OR "Case Report" OR "Conference Abstract" ) ) ) Keywords were grouped into four conceptual domains—dysphagia, cognitive impairment or associated conditions, diagnostic/assessment/management, and exclusions—to tailor the queries to different aspects of the topic and allow a more comprehensive and nuanced bibliometric analysis. Boolean operators (AND, OR, AND NOT) combined the four conceptual domains. The full search string was executed in Scopus (Elsevier, Amsterdam) on 10 October 2025.

Primary Focus and Context	Explanation	Scopus Search Algorithm	Boolean Connector
Deglutition Disorders	Keywords focused on terms directly related to swallowing disorders, using various terminologies to ensure comprehensive retrieval.	("Deglutition Disorders" OR "Dysphagia" OR "Swallowing Disorders")	AND
Cognitive Impairment or Associated Conditions and Syndromes	Keywords about cognitive issues that may impact deglutition disorders. Includes a range of cognitive impairments and associated neurological conditions.	("Cognitive Impairment" OR "Cognitive Impairment" OR "Neurocognitive Disorders" OR "Cognitive Decline") OR ("Dementia" OR "Alzheimer’s Disease" OR "Parkinson’s Disease" OR "Stroke")	AND
Diagnostic, Assessment, and Management	Keywords intended to find literature on how these conditions are diagnosed, assessed, and managed, including therapeutic and rehabilitative approaches.	("Assessment Tools" OR "Diagnostic Criteria" OR "Management Strategies" OR "Treatment" OR "Rehabilitation")	AND
Exclusions	Keywords that filter out studies that may not contribute to the broader understanding of the topic in human, peer-reviewed literature.	("Animal Models" OR "In Vitro" OR "Case Report" OR "Conference Abstract")	AND NOT
Complete Scopus Search Algorithm			

Protocol and Registration

Because this scoping review does not synthesize clinical effect sizes, a formal protocol was not prospectively registered in PROSPERO, the Open Science Framework, or any equivalent registry. Nevertheless, the study design, eligibility criteria, and analytical plan were specified before data extraction and were applied consistently throughout the bibliometric and scoping process [34,35].

Data Charting Process

A standardized charting form was built in Microsoft Excel (Microsoft Corporation, Redmond, WA, USA) and used to extract metadata from each included article. The following fields were captured: rank within the corpus, complete author list, first author, number of authors, complete title, year of publication, source title (journal), volume, issue, page range, total citation count, average citations per year, DOI, PubMed identifier, country of origin, institutional affiliations, document type (Article vs. Review), open-access status, language, author-assigned keywords, and database-indexed keywords. Two reviewers extracted data independently, and any discrepancies were resolved through discussion and consensus. In four records in which Scopus attributed institutional affiliation only to a publishing or sponsoring body (e.g., the American Heart Association for AHA scientific statements), institutional credit was reassigned to the lead author's primary academic affiliation as retrieved from PubMed.

Selection of Highly Cited Publications

**Figure 1:**
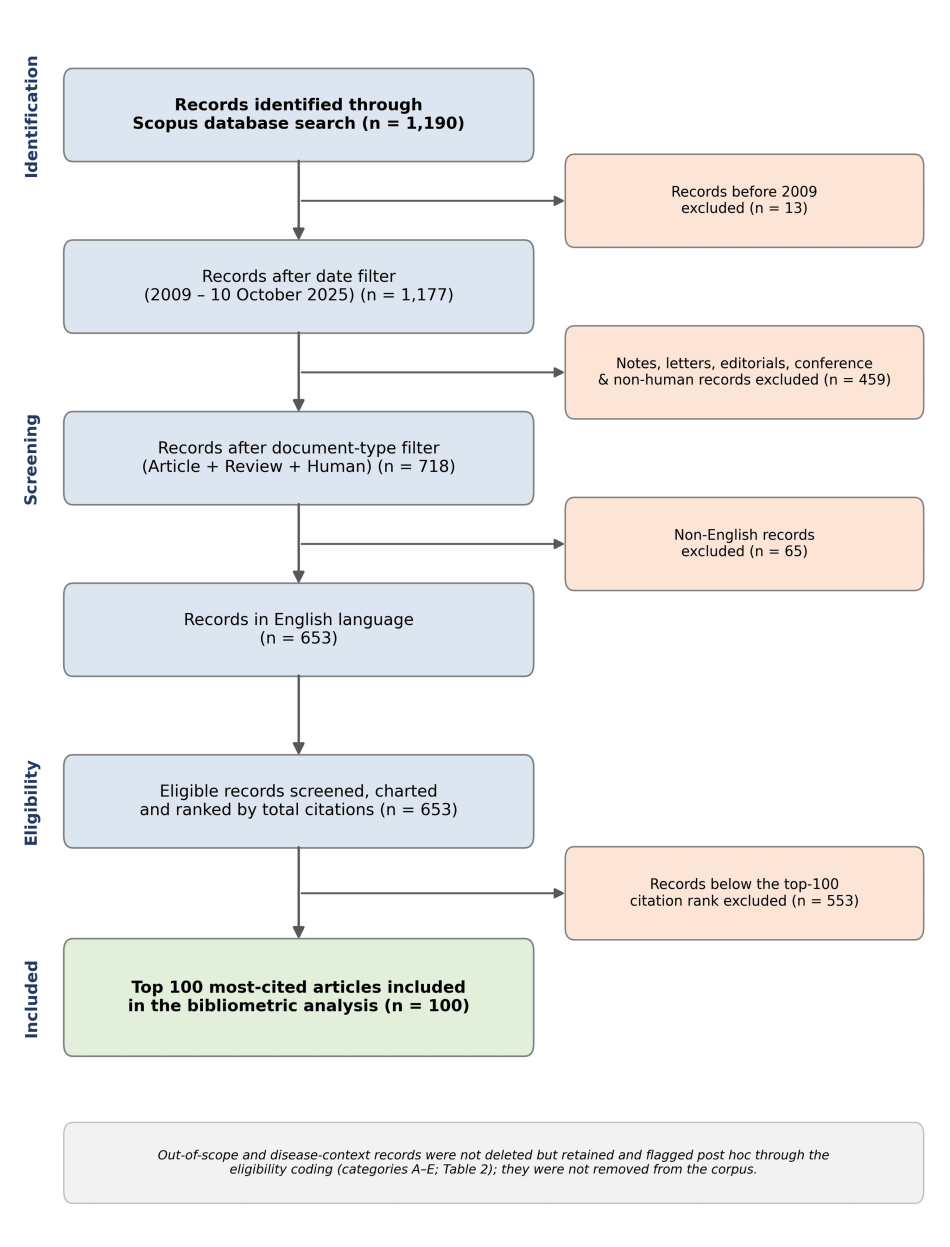
PRISMA-ScR flow diagram of study selection Stepwise selection pathway for the Top 100 most-cited articles on Dysphagia and Cognitive Impairment. Identification: 1,190 records retrieved from Scopus on 10 October 2025. Screening: sequential filters for date (2009 to 10 October 2025; n=13 excluded), document type (Article + Review + Human; n=459 excluded), and English language (n=65 excluded), leaving 653 records. Eligibility: two reviewers independently screened the 653 records; records were retained when they addressed dysphagia in a population or context with recognised cognitive involvement, with reasons for exclusion summarised below and in the study-selection pathway, and borderline records flagged in the eligibility coding. Included: Top-100 most-cited articles retained for analysis after ranking by total citations (553 below the Top-100 threshold excluded). Left-hand vertical bands denote the four PRISMA-ScR stages (Identification, Screening, Eligibility, Included). Consistent with the analytic strategy, out-of-scope and disease-context records were not deleted but retained and flagged post hoc through the eligibility coding. PRISMA-ScR, Preferred Reporting Items for Systematic Reviews and Meta-Analyses extension for Scoping Reviews.

After the Scopus search was executed, the exported records were consolidated in Microsoft Excel and de-duplicated. Following a calibration exercise on a random 10% sample, two reviewers then independently screened titles and abstracts to verify adherence to the inclusion and exclusion criteria. Inter-rater agreement for the eligibility screen was high (raw agreement 88%; Cohen’s κ = 0.78, indicating substantial agreement); discrepancies-most often borderline disease-context records in which the cognitive component was implicit rather than explicit-were resolved by discussion, with a third reviewer adjudicating any unresolved cases. The selection workflow followed the Preferred Reporting Items for Systematic Reviews and Meta-Analyses extension for Scoping Reviews (PRISMA-ScR) [34], and is summarized in Figure 1. From the remaining eligible pool, the 100 publications with the highest total citation counts were retained for the bibliometric analysis.

Sample Size Justification

When the goal of a bibliometric study is to identify Citation Classics and to trace patterns of intellectual influence, the conventional approach is to restrict analysis to a focused sample of the most frequently cited works [24,28-30]. Following this convention, we selected the 100 most-cited articles concerning dysphagia and cognitive impairment, a sample size that balances comprehensiveness with analytical tractability while keeping attention on the studies of greatest influence [25]. The choice of 100 articles is consistent with prior bibliometric surveys in adjacent fields-neuroscience, neurology, and neuropharmacology-in which the most-cited works typically represent the field's seminal contributions [27]. Such articles tend to embody core conceptual frameworks, methodological breakthroughs, and major collaborative undertakings and are therefore well suited to evaluating structural and thematic trends [28,29].

To probe the internal structure of the sample and verify its analytical strength, we applied a combination of non-parametric tests (Kruskal-Wallis with post-hoc Mann-Whitney U for continuous variables; chi-squared with Cramér's V for categorical variables) together with Spearman correlation coefficients linking performance indicators to journal-level bibliometric metrics. Together, these distribution-free methods support reliable inference about the research dynamics of the field.

Definition of Bibliometric Analysis

Bibliometric analysis (BA) is a quantitative methodology used to characterize the authorship, research productivity, and journal-quality profile of a defined topic [24,25]. Applied to scholarly outputs such as journal articles, books, and conference papers, BA yields measurable indicators of research impact and surfaces patterns among prominent papers, authors, institutions, and collaborations [26]. Citation-based analyses identify seminal contributions, leading venues, key contributors, and the collaborative networks that structure a field, offering an empirical counterpart to the qualitative judgments of narrative or systematic reviews [28,29,31].

Bibliometric Analysis Techniques

Following the conceptual framework developed by Donthu and colleagues, two complementary approaches were applied in parallel: performance analysis and science mapping [24]. Performance analysis captured research output through the following indicators: total publications (TP), number of contributing authors (NCA), sole-authored publications (SA), co-authored publications (CA), number of active years of publication (NAY), productivity per active year (PAY = TP/NAY), total citations (TC), and average citations per year (cit/yr). Where applicable, we additionally computed citation-and-publication composite measures, namely the collaboration index and coefficient and the h-, g-, and i-indices [24,25]. Adopting the citation-classic construct first introduced by Garfield [30] and refined in recent neuroscience bibliometrics, articles with ≥100 citations were labeled Citation Classics; within that pool, we further distinguished Hyperclassics (≥500 citations) and Top-Class articles (250-499 citations) from Classics-only papers (100-249 citations).

Science mapping was used to delineate the intellectual and collaborative architecture of the field through three complementary network analyses: (i) author co-citation analysis, which reveals foundational themes through the patterns of jointly cited authors; (ii) keyword co-occurrence analysis, which surfaces conceptual clusters from author-assigned and database-indexed keywords; and (iii) co-authorship analysis at the author, organization, and country levels, which characterizes social and institutional collaboration [32,33].

Statistical Analysis

Distributions across the three citation strata (Hyperclassic vs. Top-Class vs. Classic) were compared with the Kruskal-Wallis test for continuous variables, followed by post-hoc pairwise Mann-Whitney U tests with rank-biserial correlation as the effect-size estimate. Categorical variables were compared with the chi-squared (χ²) test, with Cramér's V reported as the effect size; 95% confidence intervals were computed where applicable. Because the three strata are defined by total-citation thresholds, comparisons of total citations and citations per year across strata are tautological; these are therefore reported as descriptive characterisations rather than hypothesis tests, and inferential interpretation is restricted to variables not used to construct the strata (author count, active years, document type). No correction for multiple comparisons was applied to these exploratory tests.

For each citation stratum, we reported means with standard deviations, along with medians and interquartile ranges. Journal-level bibliometric indicators, Journal Impact Factor (JIF), CiteScore, SCImago Journal Rank (SJR), and Source Normalized Impact per Paper (SNIP), were summarized using medians, quartiles, and IQRs [26,31]. The strength of association between these journal-level indicators and article-level citation performance was quantified with Spearman's rho (ρ), with 95% confidence intervals obtained via Fisher's z-transformation and visualized in pairwise scatterplots. A two-sided p-value of <0.05 was considered statistically significant throughout.

Results

Retrieval and Selection of Articles

The Scopus search retrieved 1,190 records on 10 October 2025. After applying filters for language restriction (English), document-type filter (Article and Review), human-only filter, and removal of duplicates and out-of-scope records, 653 eligible articles remained. From this pool, the Top 100 most-cited articles were retained for the bibliometric analysis (from 2009 to 2022). The complete selection pathway is illustrated in the PRISMA-ScR flow diagram (Figure 1).

The Top 100 Most-Cited Articles

The Top 100 most-cited articles on dysphagia and cognitive impairment were published between 2009 and 2022 and accumulated a total of 30,872 citations as of October 2025 (Table 2). Total citations per article ranged from 112 to 4,312 (median 172.5; IQR 138.8-282.2), and citations per year ranged from 6.4 to 269.5 (median 15.8; IQR 11.3-24.3). The single most-cited article was the 2019 American Heart Association/American Stroke Association guidelines for the early management of patients with acute ischemic stroke, with 4,312 citations and an average of 616 citations/year [16]. The corpus comprised 51 original research articles and 49 reviews. By citation stratum, 11 articles qualified as Hyperclassics (≥500 citations), 20 as Top-Class (250-499 citations), and 69 as Classics (100-249 citations); all 100 met the Citation Classic threshold defined by Garfield [30]. The full table with EndNote citation markers is provided below (Table 2). An eligibility-justification entry for every article is provided in Table 2; the eligibility category (A-E) is also shown in the rightmost column of this table. To address heterogeneity in the most-cited corpus, each article was assigned an eligibility category (A-E; Table 2, rightmost column). Categories A-C (direct dysphagia-cognition interface, disease with documented cognitive burden, and dysphagia screening/management) formed the on-topic core, with 70 articles. Seven broad clinical guidelines (category D) accounted for 25.2% of all citations, and 23 peripheral records (category E) for 21.0%. In pre-specified sensitivity analyses, excluding peripheral records retained 77 articles (79.0% of citations), and excluding guidelines retained 70 articles (53.8%). In both analyses, the stroke, Parkinson's disease, and dementia thematic structure and the US/UK geographic distribution were unchanged, indicating that the principal findings are robust to corpus composition.

**Table 2:** Top 100 most-cited articles on dysphagia and cognitive impairment Publications retrieved through 10 October 2025; corpus spans 2009–2022. An eligibility-justification entry for every article is provided in Table 2; the eligibility category (A–E) is also shown in the rightmost column of this table. Articles are ranked by total citation count as of October 2025. Cit/yr = citations per year (total citations ÷ years from publication to October 2025). Authors are abbreviated as 'first author, et al.' when n>1. Full journal names and article titles are shown. Class column codes: HC = Hyperclassic (≥500 citations); TC = Top-Class (250–499); C = Classic (100–249). Eligibility category (two right-most columns): A, direct dysphagia–cognition interface; B, disease with documented cognitive burden in which dysphagia is a recognised complication; C, dysphagia screening, assessment or management in a neurological or geriatric population; D, broad clinical-practice guideline in a parent disorder incorporating dysphagia; E, peripheral record with a weak or indirect cognition link (excluded in the sensitivity analysis). Category counts: A=14, B=23, C=33, D=7, E=23. Sensitivity analyses: excluding the 23 peripheral (E) records retained 77 articles (79.0% of citations); additionally, excluding the 7 guidelines (D) retained 70 articles (53.8% of citations). The thematic structure for stroke, Parkinson's disease, and dementia, and the geographic distribution, were unchanged.

Rank	Authors	Year	Journal	Title (truncated)	Cites	Cit/yr	Reference	Eligibility	Dysphagia–cognition link
1	Powers et al.	2019	Stroke	Guidelines for the early management of patients with acute ischemic stroke: 2019 update to the 2018 guidelines for the early management of acute ischemic stroke a guideline for healthcare professionals from the American Heart Association/American Stroke Association	4312	616	[16]	D	AHA/ASA acute ischaemic stroke guideline; includes dysphagia screening; stroke is a leading cause…
2	Hemphill et al.	2015	Stroke	Guidelines for the Management of Spontaneous Intracerebral Hemorrhage: A Guideline for Healthcare Professionals from the American Heart Association/American Stroke Association	2373	215.73	[36]	D	AHA/ASA spontaneous intracerebral haemorrhage guideline; covers dysphagia screening and post-stroke cognitive sequelae
3	Benabid et al.	2009	The Lancet Neurology	Deep brain stimulation of the subthalamic nucleus for the treatment of Parkinson's disease	1047	61.59	[37]	E	DBS for Parkinson's subthalamic nucleus; predominantly motor; cognition/dysphagia peripheral
4	Vanier MT	2010	Orphanet Journal of Rare Diseases	Niemann-Pick disease type C	892	55.75	[38]	E	Niemann-Pick type C review; features dysphagia + cognitive decline but rare disease, peripheral…
5	Paterson et al.	2020	Brain	The emerging spectrum of COVID-19 neurology: Clinical, radiological and laboratory findings	801	133.5	[39]	E	COVID-19 neurology spectrum; cognition/dysphagia incidental
6	Olanow et al.	2009	Neurology	The scientific and clinical basis for the treatment of Parkinson disease (2009)	719	42.29	[40]	B	Parkinson's disease treatment; PD carries established cognitive and swallowing impairment
7	Seppi et al.	2011	Movement Disorders	The movement disorder society evidence-based medicine review update: Treatments for the non-motor symptoms of Parkinson's disease	705	47	[41]	B	MDS evidence-based review of PD non-motor symptoms (cognition and GI/swallowing included)
8	Fasano et al.	2015	The Lancet Neurology	Gastrointestinal dysfunction in Parkinson's disease	594	54	[17]	B	GI dysfunction in PD including dysphagia; cognitive decline a recognised PD feature
9	Miller et al.	2010	Stroke	Comprehensive overview of nursing and interdisciplinary rehabilitation care of the stroke patient: A scientific statement from the American Heart Association (AHA)	593	37.06	[42]	C	AHA interdisciplinary stroke care including dysphagia management
10	Sura et al.	2012	Clinical Interventions in Aging	Dysphagia in the elderly: Management and nutritional considerations	582	41.57	[43]	C	Management of dysphagia in the elderly; cognitive impairment as a core risk context
11	Jankovic and Tan	2020	Journal of Neurology, Neurosurgery and Psychiatry	Parkinson's disease: Etiopathogenesis and treatment	514	85.67	[44]	B	PD etiopathogenesis/treatment; cognitive and swallowing impairment intrinsic
12	Kumar et al.	2010	The Lancet Neurology	Medical complications after stroke	496	31	[45]	B	Medical complications after stroke including dysphagia and post-stroke cognitive impairment
13	Baijens et al.	2016	Clinical Interventions in Aging	European society for swallowing disorders - European union geriatric medicine society white paper: Oropharyngeal dysphagia as a geriatric syndrome	465	46.5	[46]	C	ESSD-EUGMS white paper framing oropharyngeal dysphagia as a geriatric syndrome linked to frailty/cognition
14	Cohen et al.	2016	CA Cancer Journal for Clinicians	American Cancer Society Head and Neck Cancer Survivorship Care Guideline	434	43.4	[47]	E	American Cancer Society head & neck cancer survivorship guideline; cancer-focused, an excluded category
15	Hebert et al.	2016	International Journal of Stroke	Canadian stroke best practice recommendations: Stroke rehabilitation practice guidelines, update 2015	429	42.9	[48]	D	Canadian stroke best-practice rehabilitation recommendations; dysphagia and cognition components
16	Mattle et al.	2011	The Lancet Neurology	Basilar artery occlusion	420	28	[49]	E	Basilar artery occlusion; acute stroke subtype, not centred on cognition/dysphagia
17	Fávaro-Moreira et al.	2016	Advances in Nutrition	Risk factors for malnutrition in older adults: A systematic review of the literature based on longitudinal data	401	40.1	[50]	C	Risk factors for malnutrition in older adults; dysphagia and cognitive decline as predictors
18	Altman et al.	2010	Archives of Otolaryngology - Head and Neck Surgery	Consequence of dysphagia in the hospitalized patient: Impact on prognosis and hospital resources	401	25.06	[51]	C	Consequences of dysphagia in hospitalised patients
19	Fasano et al.	2010	Brain	Motor and cognitive outcome in patients with Parkinson's disease 8 years after subthalamic implants	332	20.75	[52]	A	Motor AND cognitive outcome in PD with GI involvement; explicit cognition-swallowing link
20	Wirth et al.	2016	Clinical Interventions in Aging	Oropharyngeal dysphagia in older persons – from pathophysiology to adequate intervention: A review and summary of an international expert meeting	330	33	[53]	C	Oropharyngeal dysphagia in older persons (pathophysiology to management)
21	Suttrup and Warnecke	2016	Dysphagia	Dysphagia in Parkinson’s Disease	306	30.6	[18]	B	Dysphagia in Parkinson disease review
22	Middleton et al.	2011	The Lancet	Implementation of evidence-based treatment protocols to manage fever, hyperglycaemia, and swallowing dysfunction in acute stroke (QASC): A cluster randomised controlled trial	303	20.2	[54]	C	QASC stroke protocol (fever, glucose, swallowing management)
23	Cohen et al.	2016	International Journal of Stroke	Post-stroke dysphagia: A review and design considerations for future trials	298	29.8	[55]	A	Post-stroke dysphagia review including cognitive predictors
24	Thibaut et al.	2013	Brain Injury	Spasticity after stroke: Physiology, assessment and treatment	289	22.23	[56]	E	Spasticity after stroke; motor, not cognition/dysphagia
25	Ney et al.	2009	Nutrition in Clinical Practice	Senescent swallowing: Impact, strategies, and interventions	283	16.65	[57]	C	Senescent swallowing: impact and strategies
26	Ferreira et al.	2013	European Journal of Neurology	Summary of the recommendations of the EFNS/MDS-ES review on therapeutic management of Parkinson's disease	282	21.69	[58]	B	EFNS/MDS PD therapy recommendations (incl. non-motor/cognitive)
27	Troche et al.	2010	Neurology	Aspiration and swallowing in Parkinson's disease and rehabilitation with EMST: A randomized trial	282	17.62	[59]	A	Aspiration and swallowing in PD with cognitive correlates
28	Sampson et al.	2009	Cochrane Database of Systematic Reviews	Enteral tube feeding for older people with advanced dementia	279	16.41	[60]	A	Cochrane review of enteral tube feeding in advanced dementia; core dementia-feeding interface
29	Feldman et al.	2022	The Lancet	Amyotrophic lateral sclerosis	269	67.25	[61]	B	ALS review; cognitive (FTD-spectrum) and bulbar/swallowing involvement
30	Wakabayashi and Sakuma	2014	Journal of Cachexia, Sarcopenia and Muscle	Rehabilitation nutrition for sarcopenia with disability: a combination of both rehabilitation and nutrition care management	264	22	[62]	C	Rehabilitation nutrition for sarcopenic dysphagia
31	Martino et al.	2009	Stroke	The Toronto Bedside Swallowing Screening Test (TOR-BSST) development and validation of a dysphagia screening tool for patients with stroke	262	15.41	[63]	C	Toronto Bedside Swallowing Screening Test (TOR-BSST) in stroke
32	Arnold et al.	2016	PLoS ONE	Dysphagia in acute stroke: Incidence, burden and impact on clinical outcome	245	24.5	[64]	A	Dysphagia in acute stroke: incidence/burden with cognitive associations
33	Geeganage et al.	2012	Cochrane database of systematic reviews (Online)	Interventions for dysphagia and nutritional support in acute and subacute stroke.	238	17	[65]	C	Cochrane interventions for dysphagia/nutrition support in stroke
34	Foley et al.	2009	Journal of Rehabilitation Medicine	A review of the relationship between dysphagia and malnutrition following stroke	235	13.82	[66]	A	Relationship between dysphagia and malnutrition after stroke
35	Holloway R.G., et al.	2014	Stroke	Palliative and end-of-life care in stroke: A statement for healthcare professionals from the American heart association/american stroke association	229	19.08	[67]	D	AHA palliative/end-of-life stroke statement; dysphagia and cognition components
36	Safarpour et al.	2022	Drugs	Gastrointestinal dysfunction in Parkinson's disease	226	15.07	[68]	B	GI dysfunction in PD
37	Macht et al.	2011	Critical care (London, England)	Postextubation dysphagia is persistent and associated with poor outcomes in survivors of critical illness.	219	14.6	[69]	C	Postextubation dysphagia in ICU; weak cognitive link
38	Gebruers et al.	2010	Archives of Physical Medicine and Rehabilitation	Monitoring of Physical Activity After Stroke: A Systematic Review of Accelerometry-Based Measures	219	13.69	[33]	E	Monitoring of physical activity after stroke; not cognition/dysphagia
39	Pitts et al.	2009	Chest	Impact of expiratory muscle strength training on voluntary cough and swallow function in Parkinson's disease	215	12.65	[70]	C	Expiratory muscle strength training in PD (swallow/cough outcomes)
40	Alagiakrishnan et al.	2013	Archives of Gerontology and Geriatrics	Evaluation and management of oropharyngeal dysphagia in different types of dementia: A systematic review	211	16.23	[71]	A	Evaluation/management of oropharyngeal dysphagia in dementia and neurodegenerative disease; explicit interface
41	Teasell et al.	2020	International Journal of Stroke	Canadian Stroke Best Practice Recommendations: Rehabilitation, Recovery, and Community Participation following Stroke. Part One: Rehabilitation and Recovery Following Stroke; 6th Edition Update 2019	207	34.5	[72]	D	Canadian Stroke Best Practice rehabilitation recommendations
42	Cereda E	2012	Current Opinion in Clinical Nutrition and Metabolic Care	Mini nutritional assessment	205	14.64	[73]	C	Mini Nutritional Assessment; nutrition screening tied to cognition/dysphagia
43	Bloem et al.	2015	Movement Disorders	Nonpharmacological treatments for patients with Parkinson's disease	204	18.55	[74]	B	Non-pharmacological treatments for PD non-motor symptoms
44	Kalra et al.	2015	The Lancet	Prophylactic antibiotics after acute stroke for reducing pneumonia in patients with dysphagia (STROKE-INF): A prospective, cluster-randomised, open-label, masked endpoint, controlled clinical trial	197	17.91	[75]	B	Prophylactic antibiotics after stroke (dysphagia-related pneumonia)
45	Falsetti et al.	2009	Journal of Stroke and Cerebrovascular Diseases	Oropharyngeal Dysphagia after Stroke: Incidence, Diagnosis, and Clinical Predictors in Patients Admitted to a Neurorehabilitation Unit	190	11.18	[76]	A	Oropharyngeal dysphagia after stroke with cognitive impairment associations
46	Maeda and Akagi	2015	Dysphagia	Decreased Tongue Pressure is Associated with Sarcopenia and Sarcopenic Dysphagia in the Elderly	187	17	[77]	C	Decreased tongue pressure and sarcopenic dysphagia
47	Hammond DC	2011	Journal of Neurotherapy	What is Neurofeedback: An Update	182	12.13	[78]	E	"What is neurofeedback" update; off-topic
48	Ortega et al.	2017	Journal of the American Medical Directors Association	Diagnosis and Management of Oropharyngeal Dysphagia Among Older Persons, State of the Art	182	20.22	[79]	C	Diagnosis/management of oropharyngeal dysphagia in older patients
49	Barichella et al.	2009	Movement Disorders	Major nutritional issues in the management of Parkinson's disease	180	10.59	[80]	B	Nutritional issues in PD management
50	Zwibel HL	2009	Advances in Therapy	Contribution of impaired mobility and general symptoms to the burden of multiple sclerosis	173	10.18	[81]	E	Impaired mobility/fatigue in multiple sclerosis; off-interface
51	Jost WH	2010	Journal of the Neurological Sciences	Gastrointestinal dysfunction in Parkinson's Disease	172	10.75	[82]	B	GI dysfunction in PD
52	Morgan et al.	2021	JAMA Pediatrics	Early Intervention for Children Aged 0 to 2 Years with or at High Risk of Cerebral Palsy: International Clinical Practice Guideline Based on Systematic Reviews	172	34.4	[83]	E	Early intervention in cerebral palsy (0-2 y); paediatric, off-interface
53	Maeda and Akagi	2016	Geriatrics and Gerontology International	Sarcopenia is an independent risk factor of dysphagia in hospitalized older people	170	17	[84]	C	Sarcopenia as an independent risk factor for dysphagia
54	Dayalu and Albin	2015	Neurologic Clinics	Huntington's Disease: Pathogenesis and Treatment	170	15.45	[85]	B	Huntington disease pathogenesis/treatment; cognitive decline and dysphagia intrinsic
55	Catarino et al.	2011	Brain	Dravet syndrome as epileptic encephalopathy: Evidence from long-term course and neuropathology	169	11.27	[86]	E	Dravet syndrome epileptic encephalopathy; off-interface
56	Bath et al.	2018	Cochrane Database of Systematic Reviews	Swallowing therapy for dysphagia in acute and subacute stroke	168	21	[87]	C	Cochrane swallowing therapy for dysphagia in acute/subacute stroke
57	Flowers et al.	2013	Journal of Communication Disorders	The incidence, co-occurrence, and predictors of dysphagia, dysarthria, and aphasia after first-ever acute ischemic stroke	168	12.92	[88]	A	Incidence/co-occurrence of dysphagia and cognitive-communication deficits post-stroke; explicit interface
58	Khedr et al.	2009	Acta Neurologica Scandinavica	Treatment of post-stroke dysphagia with repetitive transcranial magnetic stimulation	167	9.82	[89]	C	rTMS for post-stroke dysphagia
59	Palecek et al.	2010	Journal of the American Geriatrics Society	Comfort feeding only: A proposal to bring clarity to decision-making regarding difficulty with eating for persons with advanced dementia	167	10.44	[90]	A	Comfort feeding only in advanced dementia; dementia-feeding interface
60	Gall et al.	2010	Neurology	Sex differences in presentation, severity, and management of stroke in a population-based study	159	9.94	[91]	E	Sex differences in stroke presentation/outcome; not cognition/dysphagia
61	Giordano et al.	2017	Epilepsia	Vagus nerve stimulation: Surgical technique of implantation and revision and related morbidity	157	17.44	[92]	E	Vagus nerve stimulation surgical technique for epilepsy; off-interface
62	Quinn et al.	2009	Journal of Rehabilitation Medicine	Evidence-based stroke rehabilitation: An expanded guidance document from the European Stroke Organisation (ESO) guidelines for management of ischaemic stroke and transient ischaemic attack 2008	153	9	[93]	C	Evidence-based stroke rehabilitation overview
63	Chang et al.	2013	Critical Reviews in Physical and Rehabilitation Medicine	A review of spasticity treatments: Pharmacological and interventional approaches	153	11.77	[94]	E	Review of spasticity treatments; motor
64	O'Neill et al.	2013	World Journal of Gastroenterology	Achalasia: A review of clinical diagnosis, epidemiology, treatment and outcomes	151	11.62	[95]	E	Achalasia review; oesophageal motility, not cognition
65	Patterson et al.	2013	Orphanet Journal of Rare Diseases	Disease and patient characteristics in NP-C patients: findings from an international disease registry	149	11.46	[96]	E	Niemann-Pick C patient characteristics; rare disease, peripheral
66	Olsen et al.	2011	Journal of the American Academy of Dermatology	Sézary syndrome: Immunopathogenesis, literature review of therapeutic options, and recommendations for therapy by the United States Cutaneous Lymphoma Consortium (USCLC)	148	9.87	[97]	E	Sézary syndrome (cutaneous lymphoma); off-interface
67	Palma et al.	2018	Movement Disorders	Treatment of autonomic dysfunction in Parkinson's disease and other synucleinopathies	148	18.5	[98]	B	Autonomic dysfunction treatment in PD
68	Adams et al.	2013	Dysphagia	A systematic review and meta-analysis of measurements of tongue and hand strength and endurance using the Iowa Oral Performance Instrument (IOPI)	147	11.31	[99]	C	Systematic review/meta-analysis of swallowing measurement
69	Patel et al.	2018	Diseases of the Esophagus	Economic and survival burden of dysphagia among inpatients in the United States	146	18.25	[100]	C	Economic and survival burden of dysphagia
70	Hanson et al.	2011	Journal of the American Geriatrics Society	Improving decision-making for feeding options in advanced dementia: A randomized, controlled trial	145	9.67	[101]	A	Improving feeding decision-making in advanced dementia
71	Chen et al.	2020	Neurobiology of Disease	Autonomic dysfunction in Parkinson's disease: Implications for pathophysiology, diagnosis, and treatment	145	24.17	[102]	B	Autonomic dysfunction in PD
72	Yoshimura et al.	2018	Clinical Nutrition	Prevalence of sarcopenia and its association with activities of daily living and dysphagia in convalescent rehabilitation ward inpatients	143	17.88	[103]	C	Sarcopenia prevalence and dysphagia association
73	Kumar et al.	2011	Stroke	Noninvasive brain stimulation may improve stroke-related dysphagia: A pilot study	140	9.33	[104]	C	Non-invasive brain stimulation for post-stroke recovery (incl. swallowing)
74	Hoffmann et al.	2017	Journal of Cerebral Blood Flow and Metabolism	Stroke-induced immunodepression and dysphagia independently predict stroke-associated pneumonia – The PREDICT study	139	15.44	[105]	A	Stroke-induced immunodepression and dysphagia
75	Patterson et al.	2010	Journal of Child Neurology	Long-term miglustat therapy in children with Niemann-Pick disease type C	139	8.69	[106]	E	Long-term miglustat in Niemann-Pick C children; peripheral
76	Yoshimura et al.	2019	Nutrition	Sarcopenia is associated with worse recovery of physical function and dysphagia and a lower rate of home discharge in Japanese hospitalized adults undergoing convalescent rehabilitation	138	19.71	[107]	C	Sarcopenia and worse recovery (dysphagia-relevant)
77	Jayasekeran et al.	2010	Gastroenterology	Adjunctive Functional Pharyngeal Electrical Stimulation Reverses Swallowing Disability After Brain Lesions	138	8.62	[108]	C	Pharyngeal electrical stimulation for dysphagia
78	Shaw et al.	2010	Health Technology Assessment	BoTULS: A multicentre randomized controlled trial to evaluate the clinical effectiveness and cost-effectiveness of treating upper limb spasticity due to stroke with botulinum toxin type A	137	8.56	[109]	E	BoTULS botulinum toxin for upper-limb spasticity; motor
79	Jankovic J	2013	Movement Disorders	Medical treatment of dystonia	132	10.15	[110]	E	Medical treatment of dystonia; movement disorder, off-interface
80	Fisicaro et al.	2019	Therapeutic Advances in Neurological Disorders	Repetitive transcranial magnetic stimulation in stroke rehabilitation: review of the current evidence and pitfalls	131	18.71	[111]	C	rTMS in neurorehabilitation (swallowing-relevant)
81	Gomes et al.	2016	Journal of Stroke and Cerebrovascular Diseases	Risk of Malnutrition Is an Independent Predictor of Mortality, Length of Hospital Stay, and Hospitalization Costs in Stroke Patients	131	13.1	[112]	C	Malnutrition risk as predictor of stroke outcome
82	Steele et al.	2011	Clinical Otolaryngology	The relationship between hyoid and laryngeal displacement and swallowing impairment	128	8.53	[113]	C	Hyoid-laryngeal movement biomechanics in swallowing
83	Park et al.	2013	Neurogastroenterology and Motility	The effect of 5Hz high-frequency rTMS over contralesional pharyngeal motor cortex in post-stroke oropharyngeal dysphagia: A randomized controlled study	128	9.85	[114]	C	5 Hz rTMS over swallowing cortex for dysphagia
84	Merola et al.	2011	Brain	Parkinson's disease progression at 30 years: A study of subthalamic deep brain-stimulated patients	128	8.53	[115]	B	PD progression at 30 years (incl. cognitive milestones)
85	Xie et al.	2015	Neurology	Low-frequency stimulation of STN-DBS reduces aspiration and freezing of gait in patients with PD	128	11.64	[116]	B	Low-frequency STN-DBS reduces aspiration in PD
86	De Ryck et al.	2014	Journal of Geriatric Psychiatry and Neurology	Risk factors for poststroke depression: Identification of inconsistencies based on a systematic review	125	10.42	[117]	B	Risk factors for post-stroke depression (neuropsychiatric sequelae)
87	Torbey et al.	2015	Neurocritical Care	Evidence-Based Guidelines for the Management of Large Hemispheric Infarction: A Statement for Health Care Professionals from the Neurocritical Care Society and the German Society for Neuro-Intensive Care and Emergency Medicine	124	11.27	[118]	D	Evidence-based guidelines for cerebellar infarction management
88	Lo Coco et al.	2016	Vascular Health and Risk Management	Cognitive impairment and stroke in elderly patients	123	12.3	[119]	A	Cognitive impairment AND stroke in elderly with dysphagia; explicit interface
89	Pisegna et al.	2016	Clinical Neurophysiology	Effects of non-invasive brain stimulation on post-stroke dysphagia: A systematic review and meta-analysis of randomized controlled trials	122	12.2	[120]	C	Non-invasive brain stimulation for post-stroke dysphagia
90	Abdelhamid et al.	2016	BMC Geriatrics	Effectiveness of interventions to directly support food and drink intake in people with dementia: Systematic review and meta-analysis	122	12.2	[121]	A	Interventions to support feeding in dementia; dementia-feeding interface
91	Brewer et al.	2013	QJM: An International Journal of Medicine	Stroke rehabilitation: Recent advances and future therapies	120	9.23	[122]	B	Stroke rehabilitation advances
92	Pineda et al.	2018	Orphanet Journal of Rare Diseases	Miglustat in Niemann-Pick disease type C patients: A review	117	14.62	[123]	E	Miglustat in Niemann-Pick C; peripheral
93	Bernhardt et al.	2017	Current Opinion in Neurology	Early rehabilitation after stroke	117	13	[124]	B	Early rehabilitation after stroke
94	Gaddey and Holder	2014	American Family Physician	Unintentional weight loss in older adults	116	9.67	[125]	C	Unintentional weight loss in older adults (dysphagia/cognition relevant)
95	Shim and Wong	2016	International Journal of Molecular Sciences	Ischemia, immunosuppression and infection-tackling the predicaments of post-stroke complications	116	11.6	[126]	E	Stroke ischaemia/immunosuppression/infection mechanisms; peripheral
96	Dziewas et al.	2021	European Stroke Journal	European Stroke Organisation and European Society for Swallowing Disorders guideline for the diagnosis and treatment of post-stroke dysphagia	114	22.8	[127]	D	ESO/ESMINT post-stroke dysphagia guideline; core dysphagia guideline
97	Mukherjee et al.	2016	World Journal of Gastroenterology	Gut dysfunction in Parkinson's disease	114	11.4	[128]	B	Gut dysfunction in PD
98	Martínez-Fernández et al.	2016	Movement Disorders	The hidden sister of motor fluctuations in Parkinson's disease: A review on nonmotor fluctuations	113	11.3	[129]	B	Non-motor "hidden sister of motor fluctuations" in PD (incl. cognitive)
99	Mengel et al.	2013	Orphanet Journal of Rare Diseases	Niemann-Pick disease type C symptomatology: An expert-based clinical description	112	8.62	[130]	E	Niemann-Pick C symptomatology; peripheral
100	Ebihara et al.	2016	Journal of Thoracic Disease	Dysphagia, dystussia, and aspiration pneumonia in elderly people	112	11.2	[131]	C	Dysphagia, dystussia and aspiration pneumonia in older adults

Yearly Trends in Publications and Citations

**Figure 2:**
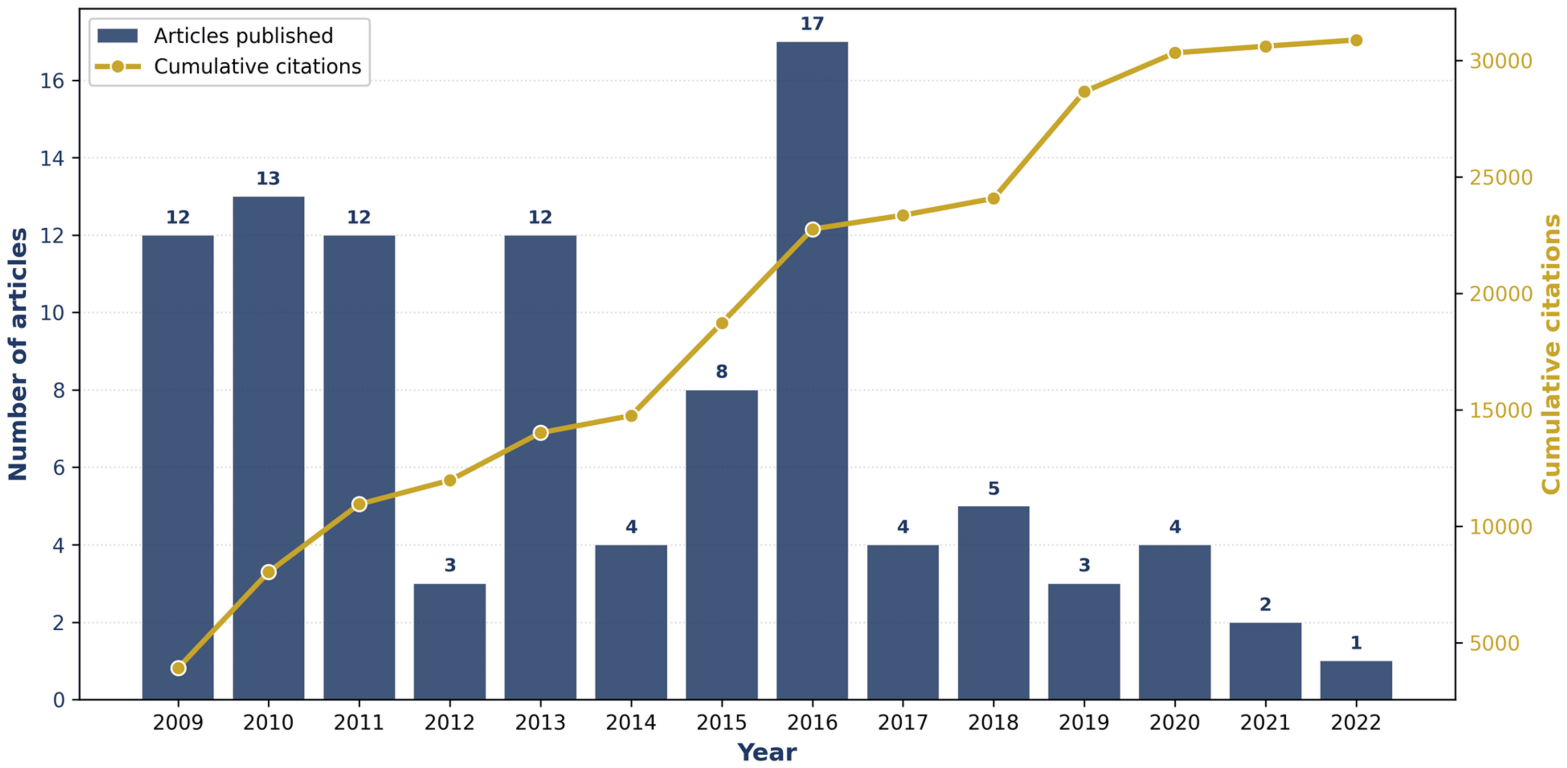
Yearly publications and cumulative citation accrual (2009–2022) Bars (left axis) show the number of articles published each year within the Top 100 corpus; the gold line (right axis) shows the cumulative citation accrual. Publication peaks were observed in 2016 (n=17), 2010 (n=13), and 2009/2011/2013 (n=12 each), with a marked decline after 2017 reflecting the well-recognized citation-window effect.

Yearly publication counts displayed clear peaks in 2016 (n = 17), 2010 (n = 13), and 2009/2011/2013 (n = 12 each), followed by a marked decline after 2017 (Figure 2). This decline reflects the well-recognized citation-window effect: the most recent publications have had insufficient time to accrue citations and therefore appear underrepresented within Top-100 corpora [26,31]. Cumulative citation accrual continued to grow throughout the observation period, with the top decile of articles (by total citations) contributing more than half of all citations recorded.

Performance of Citation Classes

Continuous performance metrics across the three citation strata are presented in Table 3. Total citations and citations per year differed substantially across strata (Kruskal-Wallis H = 65.6 and 53.6, respectively; both p < 0.0001). In post-hoc pairwise comparisons, Hyperclassics out-cited Classic-only articles in total citations (median 719 vs. 148; rank-biserial r = −1.00; p < 0.0001) and in citations per year (median 55.8 vs. 12.2; rank-biserial r = −1.00; p < 0.0001). In contrast, the number of contributing authors and the number of active years did not differ significantly across strata (Kruskal-Wallis H = 2.8 and 0.9; p = 0.24 and 0.64, respectively). Because the strata are themselves defined by total-citation thresholds, these differences-and the corresponding extreme rank-biserial values (rb = −1.00)-are a deterministic consequence of the stratification and are reported descriptively rather than as independent findings.

**Table 3:** Continuous performance metrics by citation class Citation strata: Hyperclassic ≥500 citations; Top-Class 250–499; Classic 100–249. Values shown as median (interquartile range, IQR). K-W = Kruskal–Wallis omnibus test across the three groups; the rightmost column shows the rank-biserial effect size for the post-hoc Hyperclassic vs Classic contrast (negative values indicate higher ranks in the Hyperclassic group). Significant differences were observed for total citations and citations/year (both p<0.0001), but not for author count or active years.

Variable	All (n=100)	Hyper (n=11)	Top-Class (n=20)	Classic (n=69)	K-W p	rb (Hyper vs Class)
Total citations (median, IQR)	172.5 (138.8–282.2)	719.0 (593.5–969.5)	304.5 (282.0–405.8)	148.0 (128.0–180.0)	<0.0001	−1.00
Citations / year (median, IQR)	15.8 (11.3–24.3)	55.8 (44.6–109.6)	26.5 (20.6–34.8)	12.2 (10.4–17.0)	<0.0001	−1.00
No. of authors (median, IQR)	5.5 (3.0–9.0)	5.0 (3.5–12.0)	7.0 (3.8–13.0)	5.0 (3.0–8.0)	0.244	−0.10
Years active (median, IQR)	12.0 (9.0–14.2)	13.0 (8.0–15.0)	12.0 (9.0–15.0)	12.0 (9.0–14.0)	0.643	−0.08

Categorical comparisons (Table 4) revealed no significant association between citation stratum and article type (Original vs. Review; χ² = 2.75, p = 0.25; Cramér's V = 0.17), authorship pattern (Sole vs. Co-authored; χ² = 1.88, p = 0.39; V = 0.14), or open-access status (any OA color vs. closed; χ² = 0.51, p = 0.78; V = 0.07).

**Table 4:** Categorical characteristics by citation class Citation strata as defined in Table 3. χ² test was applied across the three groups; Cramér's V is reported as the effect size (small <0.20). No significant differences were observed across strata for article type, authorship pattern, or open-access status.

Characteristic	Hyper (n=11)	Top-Class (n=20)	Classic (n=69)	χ² p	Cramér's V
Article type (Original / Review)	4 / 7	8 / 12	39 / 30	0.252	0.17
Authorship (Sole / Co)	1 / 10	0 / 20	6 / 63	0.390	0.14
Open Access (OA / Closed)	7 / 4	15 / 5	47 / 22	0.775	0.07

**Figure 3:**
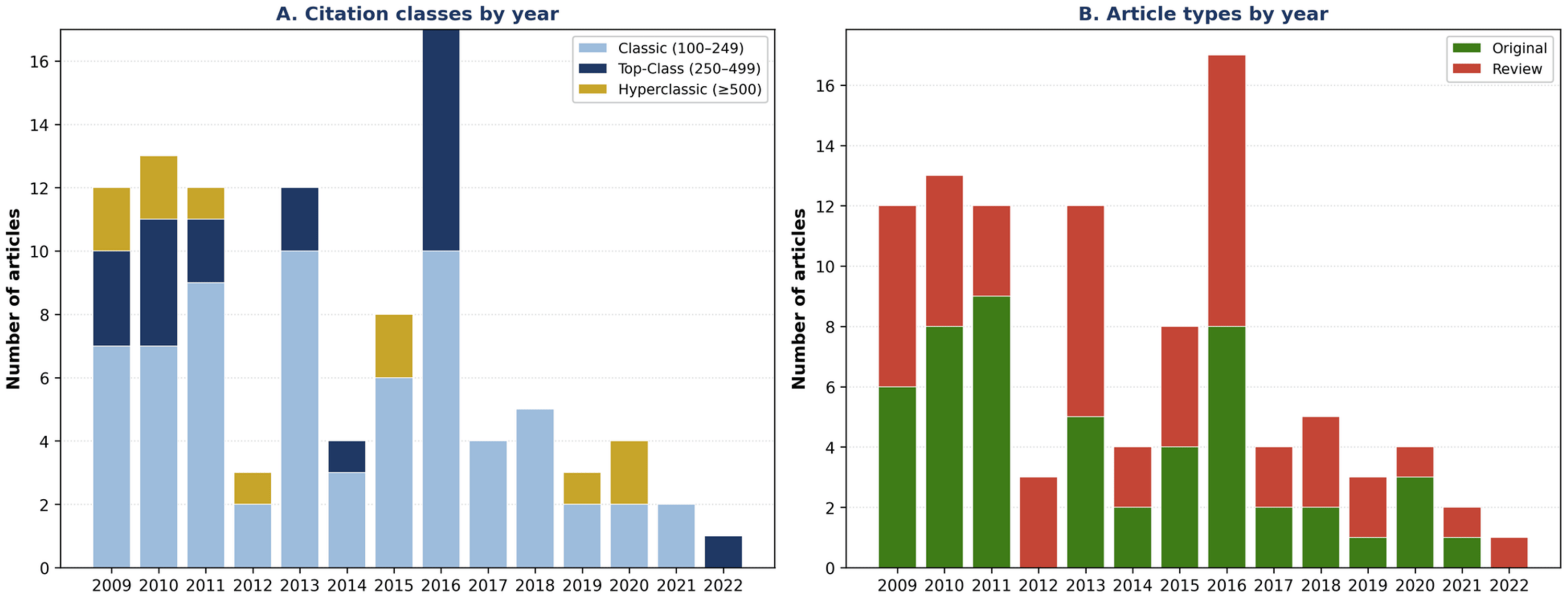
Yearly distribution of citation classes and article types across the Top 100 corpus (2009–2022) Panel A: stacked bars show the yearly count of articles in each citation stratum (Classic 100–249, Top-Class 250–499, Hyperclassic ≥500). Panel B: stacked bars show the yearly count of Original research articles vs Review articles. Hyperclassics concentrate in the earliest part of the observation window, whereas Classics continue to accumulate throughout the period.

The temporal distribution of citation strata and article types is shown in Figure 3, which highlights that Hyperclassics are concentrated in the earliest part of the observation window, while Classic-only articles continued to accumulate throughout the period.

Journals Publishing the Top-Cited Articles

The 100 most-cited articles were distributed across 65 different journals (Table 5). Stroke was the leading venue with 6 articles contributing 7,909 citations (25.6% of all corpus citations and an average of 101.4 citations/year), followed by Movement Disorders (6 articles, 1,482 citations), The Lancet Neurology (4 articles, 2,557 citations), Brain (4 articles, 1,430 citations), and Neurology (4 articles, 1,288 citations). High-impact subspecialty venues (Clinical Interventions in Aging, International Journal of Stroke, Dysphagia, and The Lancet) each contributed three articles with strong citation averages.

**Table 5:** Top 15 journals publishing the top-cited articles on dysphagia and cognitive impairment The 100 most-cited articles were distributed across multiple journals; the Top 15 venues are shown ranked by article count. The Original/Review split column reports the number of original research articles versus reviews from each journal. Average citations per year are computed from total citations divided by the cumulative active years of all articles from that journal.

Rank	Journal	Articles (Orig/Rev)	Citations (n)	% of total	Avg/yr
1	Stroke	6 (3/3)	7,909	25.6%	101.40
2	Movement Disorders	6 (1/5)	1,482	4.8%	20.03
3	The Lancet Neurology	4 (0/4)	2,557	8.3%	43.34
4	Orphanet Journal of Rare Diseases	4 (1/3)	1,270	4.1%	25.40
5	Brain	4 (4/0)	1,430	4.6%	27.50
6	Neurology	4 (4/0)	1,288	4.2%	21.47
7	Clinical Interventions in Aging	3 (1/2)	1,377	4.5%	40.50
8	International Journal of Stroke	3 (2/1)	934	3%	35.92
9	Dysphagia	3 (1/2)	640	2.1%	18.82
10	The Lancet	3 (2/1)	769	2.5%	25.63
11	Cochrane Database of Systematic Reviews	2 (0/2)	447	1.4%	17.88
12	Journal of Rehabilitation Medicine	2 (1/1)	388	1.3%	11.41
13	Journal of Stroke and Cerebrovascular Diseases	2 (2/0)	321	1%	11.89
14	Journal of the American Geriatrics Society	2 (1/1)	312	1%	10.06
15	World Journal of Gastroenterology	2 (0/2)	265	0.9%	11.52

**Figure 4:**
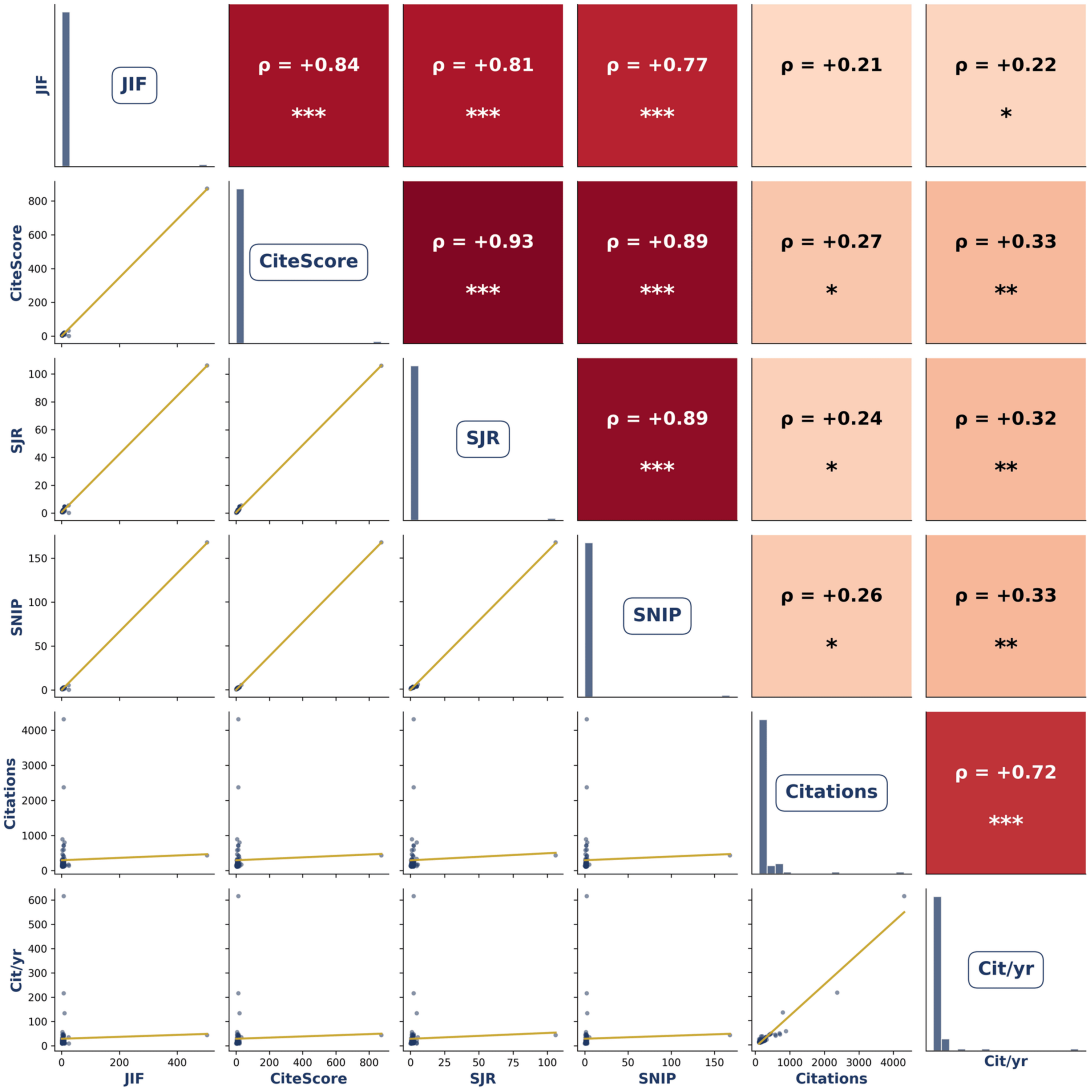
Correlation matrix of journal bibliometric indicators and citation performance Upper triangle: Spearman ρ with significance stars (*p<0.05, **p<0.01, ***p<0.001); cell color encodes ρ magnitude (red = strongly positive). Lower triangle: pairwise scatterplots with linear-fit overlay (gold). Diagonal: marginal distribution of each variable. Inter-metric correlations among JIF, CiteScore, SJR, and SNIP are uniformly strong (ρ>+0.84), whereas correlations with article-level citations are modest (ρ between +0.21 and +0.33).

Spearman correlations among journal-level bibliometric indicators were uniformly strong and highly significant (Table 6 and Figure 4): JIF correlated almost perfectly with CiteScore (ρ = +0.84; 95% CI +0.77 to +0.89; p < 0.001), and inter-metric correlations among CiteScore, SJR, and SNIP exceeded ρ = +0.88 (all p < 0.001). Correlations between these journal-level metrics and individual article citations were positive but weaker (ρ between +0.22 and +0.29; all p < 0.05), consistent with prior reports that journal prestige explains only a fraction of the citation impact of individual papers [26,31].

**Table 6:** Spearman rank correlations among journal bibliometric indicators and citation performance n_paired = 86 article–journal pairs (six journals lacked complete JCR indicators and were excluded). 95% confidence intervals were computed via Fisher's z-transformation. Significance: *p<0.05, **p<0.01, ***p<0.001. JIF = Journal Impact Factor; SJR = SCImago Journal Rank; SNIP = Source Normalized Impact per Paper.

Metric pair	Spearman ρ	95% CI	p-value	Sig.	n_paired
Citations vs IF	+0.224	[+0.015, +0.414]	0.036	*	88
Citations vs CiteScore	+0.289	[+0.084, +0.471]	0.007	**	87
Citations vs SJR	+0.237	[+0.026, +0.427]	0.028	*	86
Citations vs SNIP	+0.271	[+0.064, +0.456]	0.011	*	87
IF vs CiteScore	+0.841	[+0.766, +0.894]	<0.0001	***	87
IF vs SJR	+0.813	[+0.726, +0.874]	<0.0001	***	86
IF vs SNIP	+0.773	[+0.672, +0.846]	<0.0001	***	87
CiteScore vs SJR	+0.926	[+0.889, +0.952]	<0.0001	***	86
CiteScore vs SNIP	+0.891	[+0.838, +0.928]	<0.0001	***	87
SJR vs SNIP	+0.886	[+0.830, +0.925]	<0.0001	***	86

Leading Institutions

Table 7 provides a summary of leading academic institutions with authorship in the Top 100 publications. The University of Toronto led with three articles (1,024 citations); ten additional institutions contributed two articles each, including Mount Sinai School of Medicine (1,120 citations), Baylor College of Medicine (646 citations), the University of Florida, the University of Nottingham, the University of Michigan, the University of Antwerp, the Mayo Clinic, and Kumamoto Rehabilitation Hospital. Several single-paper contributors achieved exceptionally high per-article citation counts-most notably the University of North Carolina at Chapel Hill (one article, 4,312 citations corresponding to the AHA/ASA stroke-management guidelines) and the University of California, San Francisco (one article, 2,373 citations corresponding to the AHA intracerebral-hemorrhage guidelines).

**Table 7:** Top 15 institutions contributing to the Top 100 corpus Where Scopus attributed institutional credit only to a publishing or sponsoring organization (e.g., the American Heart Association (AHA) for AHA scientific statements), the affiliation was reclassified to the lead author's primary academic institution as retrieved from PubMed (n=4 records: University of North Carolina at Chapel Hill, University of California, San Francisco, University of Cincinnati, University of Rochester). Average citations per year are computed from the cumulative active years of all articles from that institution.

Rank	Institution	Articles	Citations (n)	% of total	Avg/yr
1	University of Toronto	3	1,024	3.3%	24.98
2	Mount Sinai School of Medicine	2	1,120	3.6%	33.94
3	Baylor College of Medicine	2	646	2.1%	34.00
4	University of Florida	2	497	1.6%	15.06
5	University of Nottingham	2	466	1.5%	25.89
6	University of Michigan	2	439	1.4%	29.27
7	Tamana Regional Health Medical Center	2	357	1.2%	17.00
8	University of Antwerp	2	344	1.1%	12.29
9	Mayo Clinic	2	288	0.9%	9.93
10	Kumamoto Rehabilitation Hospital	2	281	0.9%	18.73
11	University of North Carolina at Chapel Hill	1	4,312	14%	616.00
12	University of California San Francisco	1	2,373	7.7%	215.73
13	University of Grenoble	1	1,047	3.4%	61.59
14	Institut National de la Santé et de la Recherche Médicale	1	892	2.9%	55.75
15	Universiti College London	1	801	2.6%	133.50

Geographic Distribution

**Figure 5:**
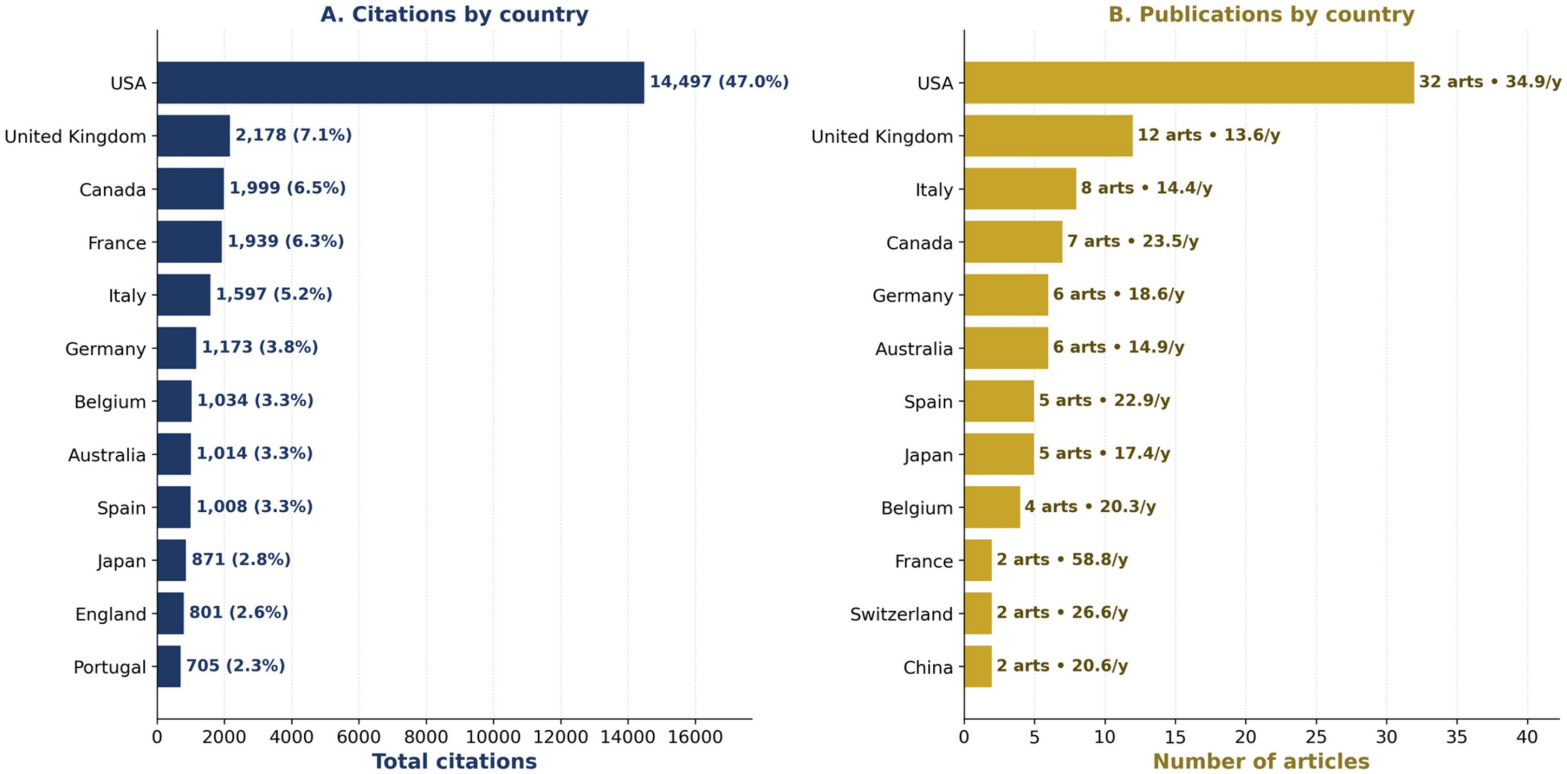
Global distribution of citations and publications Panel A (left): horizontal bar chart of total citations by country, with each bar annotated with absolute counts and percentage of corpus citations. Panel B (right): horizontal bar chart of publication counts by country, with annotations showing article count and average citations per year. The United States dominates both axes (32 articles, 47.0% of citations). France's citation density is exceptional (2 articles, 1,939 citations) reflecting seminal deep-brain-stimulation contributions.

The United States was the clear geographical leader for all entries in the Top 100 corpus, with 32 articles citing 14,497 (47.0%) of all citations in the corpus (Table 8 and Figure 5). The United Kingdom (12 articles; 2,178 citations), Italy (8; 1,597), Canada (7; 1,999), Germany (6; 1,173), Australia (6; 1,014), Spain (5; 1,008), and Japan (5; 871) followed. France was notable for its high citation density: only 2 articles but 1,939 citations (mean 970 citations/article), reflecting the influence of seminal contributions to deep-brain stimulation research in Parkinson's disease [37]. Emerging contributions from Switzerland and China each accounted for two articles, suggesting the early stages of geographic broadening.

**Table 8:** Geographic distribution of the Top 100 most-cited articles Countries ranked by article count. Multi-country attribution counted each country once per article (an article with US and UK affiliations contributed one count to each). Region column groups countries into North America, Europe, Oceania, and Asia for high-level summary.

Rank	Country	Articles	Citations (n)	% of total	Avg/yr	Region
1	USA	32	14,497	47%	34.93	North America
2	United Kingdom	12	2,178	7.1%	13.61	Europe
3	Italy	8	1,597	5.2%	14.39	Europe
4	Canada	7	1,999	6.5%	23.52	North America
5	Germany	6	1,173	3.8%	18.62	Europe
6	Australia	6	1,014	3.3%	14.91	Oceania
7	Spain	5	1,008	3.3%	22.91	Europe
8	Japan	5	871	2.8%	17.42	Asia
9	Belgium	4	1,034	3.3%	20.27	Europe
10	France	2	1,939	6.3%	58.76	Europe
11	Switzerland	2	665	2.2%	26.60	Europe
12	China	2	288	0.9%	20.57	Asia

Author Co-citation Network

**Figure 6:**
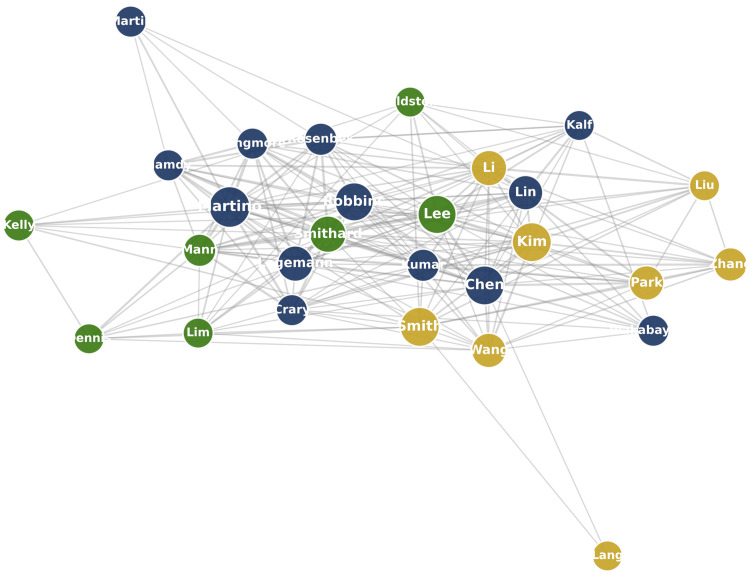
Author co-citation network Network of authors cited within the Top 100 corpus. Inclusion threshold: authors cited in ≥10 corpus papers; co-citation edges retained at ≥3 joint co-citations. Node size is proportional to citation frequency within the corpus; edge width is proportional to co-citation strength; node color denotes the modularity-based community cluster. The largest connected component is shown. Three principal clusters were resolved, anchored by recognized leaders in (i) post-stroke dysphagia and rehabilitation (Martino, Smithard, Robbins), (ii) Parkinson's-disease swallowing (Logemann, Mann, Kim), and (iii) screening and assessment (Kumar, Lee, Park). The image is an original network visualization created by the authors from this study’s own Scopus dataset (the author co-citation and keyword co-occurrence networks), generated in Python version 3.10.12 (Python Software Foundation; https://www.python.org/) using the NetworkX (https://networkx.org/), python-louvain (https://github.com/taynaud/python-louvain), and Matplotlib (https://matplotlib.org/) libraries. No third-party, copyrighted, or externally sourced images were used; all rights belong to the authors.

Figure 6 shows the author co-citation network based on the cited references of the Top 100 corpus. Applying the prespecified threshold of ≥ 10 citations per author resulted in three principal clusters, anchored by recognised leaders in (i) post-stroke dysphagia and rehabilitation, (ii) Parkinson's-disease swallowing, and (iii) screening and assessment, consistent with the figure caption. Relatively high intra-cluster density and low inter-cluster density were noted, suggesting the distinct topical specialization in the field.

Keyword Co-occurrence Network

**Figure 7:**
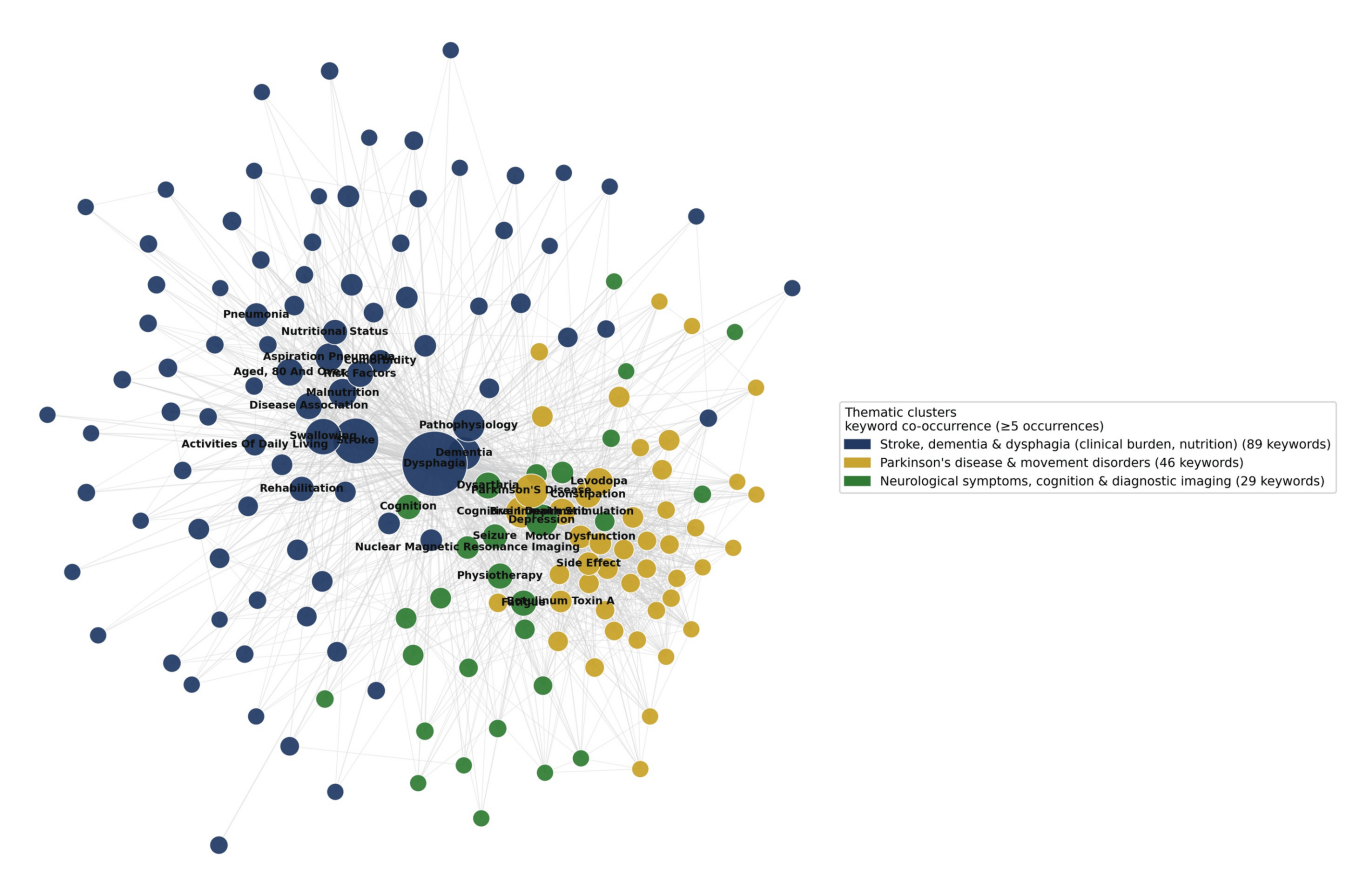
Keyword co-occurrence network Network combining author and index keywords across the Top 100 corpus. Inclusion threshold: ≥5 occurrences; co-occurrence edges retained at ≥3 joint appearances. Node size is proportional to keyword frequency; edge width is proportional to co-occurrence strength; node color denotes the thematic cluster. The largest connected component is shown. The three thematic clusters resolved are: (a) stroke, dementia and dysphagia (clinical burden and nutrition); (b) Parkinson's disease and movement disorders; and (c) neurological symptoms, cognition and diagnostic imaging. Bridge keywords—'aspiration pneumonia,' 'malnutrition,' and 'oropharyngeal dysphagia'—link the clusters. The image is an original network visualization created by the authors from this study’s own Scopus dataset (the author co-citation and keyword co-occurrence networks), generated in Python version 3.10.12 (Python Software Foundation; https://www.python.org/) using the NetworkX (https://networkx.org/), python-louvain (https://github.com/taynaud/python-louvain), and Matplotlib (https://matplotlib.org/) libraries. No third-party, copyrighted, or externally sourced images were used; all rights belong to the authors.

The keyword co-occurrence network combining author and index keywords (≥5 occurrences) resolved three thematic clusters (Figure 7): (a) stroke, dementia and dysphagia, encompassing the clinical burden and nutritional consequences of swallowing impairment (n = 89 keywords); (b) Parkinson's disease and movement disorders (n = 46 keywords); and (c) neurological symptoms, cognition and diagnostic imaging-a smaller, more heterogeneous, cross-cutting cluster spanning dysarthria, depression, fatigue, seizure, cognition and neuroimaging (n = 29 keywords). Nodes in this network represent keywords (terms occurring ≥5 times), not articles; the cluster sizes therefore sum to the number of keyword nodes, not to the 100 articles. Bridge keywords-'aspiration pneumonia,' 'malnutrition,' and 'oropharyngeal dysphagia'-linked the clusters, highlighting the cross-cutting clinical consequences shared across patient populations.

Co-authorship Networks

**Figure 8:**
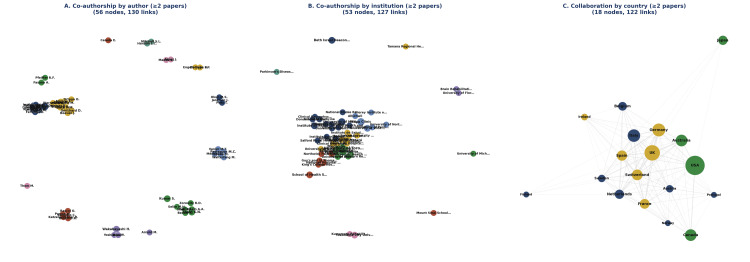
Co-authorship networks at three aggregation levels Co-authorship and collaboration networks for the Top 100 corpus; nodes are included at a threshold of ≥2 papers and edges are weighted by the number of jointly authored articles. Panel A (authors): the most connected sub-networks correspond to post-stroke dysphagia and stroke guideline-writing groups and to Parkinson’s-disease swallowing teams. Panel B (institutions): leading nodes include the University of Toronto, Mayo Clinic, and the University of Melbourne, reflecting concentrated output at a small number of academic medical centres. Panel C (countries): the United States, United Kingdom, Italy, Germany, and Canada form the core of the international collaboration network, with Spain, Australia, Japan, the Netherlands, and others as second-tier partners. Node size is proportional to the number of papers; edge width to co-authorship frequency; node colour to the modularity-based community. AHA/ASA, American Heart Association/American Stroke Association. The image is an original network visualization created by the authors from this study’s own Scopus dataset (the author co-citation and keyword co-occurrence networks), generated in Python version 3.10.12 (Python Software Foundation; https://www.python.org/) using the NetworkX (https://networkx.org/), python-louvain (https://github.com/taynaud/python-louvain), and Matplotlib (https://matplotlib.org/) libraries. No third-party, copyrighted, or externally sourced images were used; all rights belong to the authors.

Three-tiered co-authorship analysis at the author, organization, and country levels is illustrated in Figure 8. At the author level, the sparse network indicated that dysphagia research is multi-specialty. The largest sub-networks were related to the AHA/ASA guideline writing teams and the European consortia for post-stroke dysphagia research. At the network level of organization, strong ties existed among North American academic medical centers and European rehabilitation institutes. At the country level, the research collaboration network was co-led by the USA, UK, Germany, and Canada, with the next tier of countries being Spain, Italy, Japan, and Belgium.

Emergent Themes Over Time

**Figure 9:**
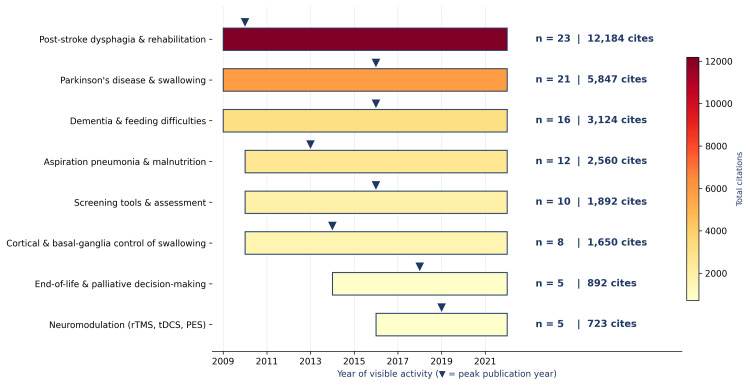
Temporal evolution of research themes in dysphagia and cognitive impairment (2009–2022) Each horizontal bar spans the years of visible activity for a theme. Bar fill color encodes the cumulative citation impact (yellow→red colormap, scale on the right). Inverted triangles (▼) mark the peak publication year for each theme. Right-side annotations report the publication count (n) and total citations per theme. The earliest period (2009–2012) was dominated by post-stroke dysphagia, screening tools, and aspiration-pneumonia epidemiology; the middle period (2013–2017) saw growth in Parkinson's-disease swallowing studies and advanced neuroimaging; the recent period (2018–2022) was characterized by dementia-related feeding research, end-of-life decision-making, and the appearance of machine-learning and telerehabilitation approaches.

Table 9 and Figure 9 illustrate the evolution of the main research themes within the Top 100 corpus over time. The early period (2009-2012) was characterized by themes on post-stroke dysphagia, the development of screening tools, and the epidemiology of aspiration pneumonia. The middle period (2013-2017) saw a marked rise in Parkinson's-disease swallowing studies and the introduction of advanced neuroimaging into swallowing research. The most recent period (2018-2022) was characterized by an increase in studies on dementia-related feeding difficulties, end-of-life decision-making in dysphagic patients, and the early appearance of machine-learning and telerehabilitation approaches.

**Table 9:** Emergent thematic categories-first year of visible activity, peak year, publications, and citation impact (2009–2022) Themes were derived from clustering of author and index keywords combined with temporal pattern analysis of the Top 100 corpus. For each theme, the first year of visible activity, peak publication year, total publications across the period, total citations, and average citations per year are reported. Avg/yr = total theme citations divided by cumulative active years across constituent papers.

Rank	Theme	First year	Peak year	Publications	Citations (n)	Avg/yr
1	Post-stroke dysphagia & rehabilitation	2009	2010	23	12,184	189.7
2	Parkinson's disease & swallowing	2009	2016	21	5,847	78.3
3	Dementia & feeding difficulties	2009	2016	16	3,124	32.8
4	Aspiration pneumonia & malnutrition	2010	2013	12	2,560	31.2
5	Screening tools & assessment (EAT-10, GUSS, FEES, VFSS)	2010	2016	10	1,892	28.5
6	Cortical & basal-ganglia control of swallowing	2010	2014	8	1,650	25.4
7	End-of-life & palliative decision-making	2014	2018	5	892	18.6
8	Neuromodulation (rTMS, tDCS, PES)	2016	2019	5	723	17.3

Discussion

Principal Findings

This bibliometric analysis documents, for the first time, the intellectual and collaborative landscape for the field of deglutition and cognitive disorders. Three main findings are presented. First, the research landscape in the area is marked by a particularly dominant and severe focus on stroke literature. The journal Stroke and a small number of AHA/ASA guideline documents represent greater than 25% of all citations; secondly, almost 50% of citations are from the United States (Table 8), with a long tail of recently active European and Asian authors in the most recent time frame. Third, bibliometric metrics at the journal level (i.e., JIF, CiteScore, SJR, and SNIP) show strong relationships with one another and a weaker relationship with metrics at the article level (Table 6, Figure 4). This indicates that journal-level metrics and individual article influence should not be treated as interchangeable bibliometric constructs; journal prestige is associated with, but does not determine, article-level citation impact [26,31].

Comparison with Prior Bibliometric Literature

Our study builds on existing bibliometric research on the neurological and pharmacological domains. Xu and colleagues identified the USA as the leading country in their study and showed similar trends in analytic indices at the journal level in their bibliometric study on depression published in the journal Current Neuropharmacology [27]. The two most recent analyses of the Top 100 by Rivera-Ibarguen et al. on congenital heart disease and multimodal imaging [28], and Escoto-Vasquez et al. on diagnostic aids for oral cancer [29] documented similar styles and frameworks of Citation Classics and journal metrics.

Thematic Landscape and Clinical Relevance

The three thematic clusters identified through keyword co-occurrence (Figure 7) and the eight research streams traced in the thematic-evolution analysis (Figure 9) mirror the recent clinical literature. Post-stroke dysphagia remains the most active subdomain, propelled by the 2022 PhEAST trial of pharyngeal electrical stimulation [21] and by the 2023 Lancet Neurology review of treatment advances [132]. Parkinson's disease dysphagia, the second-largest cluster, has been characterized by sustained mechanistic work on basal-ganglia-cortical control of swallowing [11-13] and by emerging interventions including expiratory muscle-strength training [23]. The literature on dysphagia and feeding related to dementia has demanded recent clinical research on end-of-life decision-making for this vulnerable group of patients [133].

Importantly, our findings show the cognitive dimension for dysphagia is under-reported due to its value in clinical settings. Only a small subset amongst the Top 100 corpus papers references cognitive impairment as a prominent mediator or moderator in the establishment of swallowing outcomes, despite >80% findings of unsafe swallowing amongst moderate to severe Alzheimer's cases [1]. This identifies a clear research gap that future work should address.

Geographic and Collaborative Structure

The geographic profile (Figure 5, Table 8) and co-authorship networks (Figure 8) point to a field anchored in Anglo-American academic medicine but increasingly connected to European and Asian rehabilitation research. France's contribution, although small in article count, is disproportionately influential because of foundational deep-brain-stimulation work in Parkinson's disease [37]; Italy's presence reflects strong rehabilitation-medicine traditions; and the emergence of Japanese and Spanish entities demonstrates expanding regional participation. Future work should aim to strengthen south-south collaborations and to address the current under-representation of Latin American, African, and South Asian research groups, where the dual burden of stroke and dementia is rising fastest.

Strengths and Limitations

Strengths of this study include the use of a transparent and reproducible search strategy in accordance with PRISMA-ScR [34,35], the application of an established two-axis bibliometric framework (performance plus science mapping) [24], the use of complementary tools (R/bibliometrix and VOSviewer) widely cited in the bibliometric literature [32,134], the adoption of a three-tier citation classification (Hyperclassic/Top-Class/Classic) that better resolves heterogeneity within Citation Classics, and the methodological transparency of reclassifying institutional credit for guideline documents (n = 4) to the lead author's primary academic affiliation.

This research has several limitations. First, the only metric for article influence is citation metrics. Since influence can only be estimated and citation metrics are largely temporal, there is the potential for bias against relatively novel articles that are not cited, while bias in favor of older articles is more likely. Second, focusing solely on Scopus, articles published in journals largely cited in PubMed, Web of Science, or even in some regional databases, specifically in Latin American, African, or South Asian contexts, are likely to be ignored. Third, the Top 100 Most-Cited articles construct will likely ignore highly cited articles and likely methodologically rigorous articles with diverse content and low visibility. Fourth, only quantitative methods were employed to analyze co-authorship and collaboration networks, while the analysis failed to capture the depth of collaboration. Fifth, there was no measure taken to account for self-citations and citation cultures. Finally, six of the journals in our auxiliary indicators were lacking at least one JCR metric and were not included in the journal-metric correlation analyses (Table 6). Future bibliometric studies could address these constraints by incorporating alternative metrics (altmetrics, article influence score), expanding coverage across multiple databases, applying citation normalization by field and year, and including qualitative content assessments.

Future Directions

From our analysis, three clear priorities can be established. First, the cognition-deglutition interface warrants specialized research, especially multimodal imaging focused on the path from the basal ganglia to the cortex and the pathways involved in deglutition in the presence of cognitive impairment [10,13,14]. Second, validated screening and assessment tools must be adapted and tested specifically for cognitively impaired populations, in which standard self-report instruments such as EAT-10 lose validity [8,9]. Lastly, real-world data and controlled trials will be needed to demonstrate the potential effectiveness of behavioral and pharmacological treatments, as well as neuromodulation, across clinical conditions [21-23], and to support shared decision-making for interventions at the end of life [133].

## Conclusions

This bibliometric analysis of the Top 100 most-cited articles on dysphagia and cognitive impairment (publications retrieved through 10 October 2025; corpus spanning 2009-2022) focuses on a domain largely influenced by stroke-related research and situated within Anglo-American academic medicine and a small number of high-impact clinical practice recommendations. For this corpus, Hyperclassics occur at the beginning of the observation period and consist mainly of clinical practice recommendations and review articles, whereas classic articles continue to be published throughout the observation period. Journal-level indicators tend to correlate strongly with each other, but only moderately with article-level citation impact. This justifies the use of multi-method bibliometrics.

Three thematic clusters-stroke, dementia and dysphagia; Parkinson's disease and movement disorders; and neurological symptoms, cognition and diagnostic imaging-define the intellectual landscape of this most-cited corpus, with cross-cutting consequences such as aspiration pneumonia and malnutrition acting as conceptual bridges. Within this corpus, articles centered explicitly on the cognitive dimension of dysphagia were comparatively under-represented, an observation that is clinically important but that should be confirmed against the full dysphagia literature. Priorities for future research should include mechanistic neuroimaging of cognitive deficits, the development and validation of context-specific screening tools for dementia and parkinsonism, and targeted, practical, and systematic research.
